# Inhaler devices in asthma and COPD patients – a prospective cross-sectional study on inhaler preferences and error rates

**DOI:** 10.1186/s12890-020-01246-z

**Published:** 2020-08-20

**Authors:** Jens Schreiber, Tina Sonnenburg, Eva Luecke

**Affiliations:** Department of Pneumonology, Otto-von-Guericke University, Leipziger Strasse 44, 39120 Magdeburg, Germany

**Keywords:** Asthma, COPD, Device handling, Inhaler errors, Inhaler technique, Patient preference

## Abstract

**Background:**

Inhalation therapy is the backbone of asthma and COPD control. However, inhaler adherence and device mishandling continue to be a problem in real life. Some studies have shown that using a patient-preferred inhaler may reduce device handling errors and improve adherence to prescribed chronic inhaler drug therapy. The aim of this study was to compare the preferences for commonly used inhaler devices in Germany in patients with chronic obstructive respiratory disease. We also pursued the question which properties of an inhaler device are particularly important to the user and what effects age, gender and type of disease (asthma or COPD) may have on device preference and handling errors.

**Methods:**

Prospective, open-label cross-sectional study in which 105 patients with asthma (58%) or COPD (42%) participated. Validated checklists were used to objectively assess inhaler technique and errors with 10 different placebo devices. For each device, patients were asked to test the handling, to assess the device properties and to name the device that they would most or least prefer.

**Results:**

Across the 10 placebo inhaler devices tested, patients needed an average of 1.22 attempts to error-free use. The device with the lowest mean number of attempts was the Turbohaler® (1.02), followed by the Nexthaler® (1.04), the Diskus® (1.07) and the Spiromax® (1.10). Patients over 60 years vs. younger age (*p* = 0.002) and COPD vs. asthma patients (*p* = 0.016) required more attempts to ensure correct use. 41% of the study participants chose one of the devices they already used as the most preferred inhaler. Overall, 20% opted for the Spiromax®, 15% for the Nexthaler® and 14% for the Turbohaler® or a pMDI. The least preferred device was the Elpenhaler® (0%). From a selection of 7 predefined inhaler attributes, patients stated easy handling as the most important for them. This was followed by short inhalation time and low inhalation resistance.

**Conclusions:**

Patient preference may vary between inhaler devices. The lowest number of attempts to error-free use was reported for the Turbohaler® and the Nexthaler®. The Spiromax® and the Nexthaler® achieved the best overall ratings and were the devices most preferred by patients.

## Background

Inhaled drug delivery is the cornerstone treatment for chronic respiratory disease [1]. A proper inhaler technique is therefore crucial for an effective treatment of asthma and chronic obstructive pulmonary disease (COPD) [2, 3]. In real-world, however, inadequate inhaler technique remains a problem among patients [4], and device mishandling continues to be common even in experienced patients [2]. Inhaler handling errors and non-adherence can affect drug delivery and minimize treatment benefits [5, 6]. A large number of studies reported that inhaler errors came along with worse disease outcome in asthma or COPD patients [7]. Inhaler misuse and poor adherence were associated with an increased risk of hospitalization (*p* = 0.001), emergency room visits (*p* < 0.001), oral steroids (*p* < 0.001) and antimicrobial agents (*p* < 0.001) and poor disease control [2]. In contrast, patients who achieved a reduction in errors over time had improved outcomes [7]. In addition to the error rate when using inhalers, the patient’s adherence to inhaler device therapy also plays an important role. Suboptimal adherence was associated with poor results in patients with asthma or COPD [8]. An improved usability and higher satisfaction with the device may contribute to increased patient adherence to treatment [9].

It is well known that there are many challenges with the use of inhalers, and no device suits all patients [10]. Each type of device has its own advantages and disadvantages [11]. There are three main types of devices used to deliver inhaled medication: pressurized metered-dose inhalers (pMDIs), dry powder inhalers (DPIs), and a soft mist inhaler (SMI). Each type of inhaler device is associated with advantages and limitations that determine their suitability for any given patient [12]. Understanding the pro and cons helps clinicians in choosing the proper device for the individual patient’s clinical needs and preferences. However, inhaler selection remains challenging [12].

As each inhaler offers varying technical properties, a tailored and personalized approach to the selection of the most appropriate device for the patient is highly recommended in order to increase the likelihood of achieving improved disease outcomes and enhance device adherence [10]. Choosing the most appropriate inhaler for a specific patient and regular assessment of ability to correctly use their inhaler will promote better adherence to therapy. In fact, inhaler choice is as critical as the choice of medication itself [13]. The patient’s opinions and preferences should also be taken into account when selecting the inhaler device [13, 14].

Therapeutic success of inhalation therapy therefore depends not only on the pharmacology, particle size, drug deposition and correct inhalation technique, but also on the patient’s perception, preference and satisfaction with the inhaler [8, 15]. There are several factors that determine the patient’s preference for an inhaler device. Understanding these factors is critical to improving compliance and associated treatment outcomes [8, 16, 17]. Choosing an inhaler should be a joint decision between doctor and patient, taking into account the patient’s skills, preferences, and past experience with inhalers, available medication, and cost [18].

The aim of our study was to find out which devices are favoured and what influence age and gender and medical condition have on inhaler preferences and error rates in patients with chronic obstructive respiratory disease.

## Methods

### Study design and patients

This was a prospective, open-label and industry-independent, cross-sectional study of asthma and COPD patients. Study participants were recruited from the Department of Pneumonology of the Otto-von-Guericke University Magdeburg, Germany. Both inpatients and ambulant patients were enrolled in the study. All patients were in a stable condition. Inclusion criteria comprised: a diagnosed obstructive airway disease (asthma or COPD), an age between 18 and 100 years and a signed declaration of consent to participate in the study. The patients had a several years history of the respiratory disease. There was no obvious cognitive impairment and no obvious impairment of fine motor skills. The lack of written consent was an exclusion criterion.

All patients were interviewed. We examined the inhaler technique with the patient’s own device and queried preferred inhaler attributes. Then we tested the following 10 devices that did not contain any drug (placebo inhalers) in random order:

- Dry-powder inhalers (DPI): Breezhaler®, Diskus®, Elpenhaler®, Genuair®, Nexthaler®, Forspiro®, Spiromax®, Turbohaler®
- Pressurized metered-dose inhaler (pMDI): A customary pMDI (Flutiform® pMDI) was used
- Soft mist inhaler (SMI): Respimat®

All selected devices represented approved products in Germany with a high market share. After demonstrating the use of the placebo inhalers, patients were asked to evaluate the handling and other properties of each device and to name the device that they would most or least prefer for daily use.

The study was conducted in accordance with the Declaration of Helsinki [19], and the study protocol was approved by the local Ethics Committee of the Otto-von-Guericke University Magdeburg, Germany (reference number 51/16).

### Data collection, check of inhaler technique and preferred inhaler attributes

Data was collected using a structured questionnaire that was filled in by the patient. The questionnaire was developed on the basis of the Patient Satisfaction and Preference Questionnaire (PASAPQ) and the Feeling of Satisfaction with Inhaler (FSI-10) questionnaire [20, 21]. The PASAPQ is a multi-item measure of respiratory inhalation device satisfaction, designed and validated in asthma and COPD patients [20]. The FSI-10 questionnaire evaluates the patient’s opinion regarding the ease or difficulty of using an inhaler device [21]. In order to check the comprehensibility of the questions and the practicability of the structured questionnaire used in this study, it was tested in advance in 10 patients.

After study participants had been recruited, the patient’s characteristics (gender, age, presence of asthma or COPD, disease duration) were collected first. Second, the patients specified which inhalers they currently or formerly used. Subsequently, all participants were asked to demonstrate the inhaler technique with their prescribed inhaler device for daily use. The investigator used checklists based on the recommendations of the German Respiratory League [22] to determine whether the patient’s use of their own device was correct or incorrect.

The investigator next asked which of the following predefined inhaler properties were particularly relevant to the patient: easy handling (e.g. number of steps to complete inhalation, ease to perform these steps, ease to manage the device ergonomically), short inhalation time, low inhalation resistance, discreet handling, availability of a dose counter, design and colour. Ratings were reported on a Likert scale, ranging from 1 (very important) to 4 (unimportant).

### Inhaler preferences and error rates for 10 placebo devices

The investigator then demonstrated how to use each of the 10 placebo inhaler devices. The latter were presented in a random order based on a pre-generated randomization list. The handling for each individual device was demonstrated on the basis of standardized checklists for correct use, which had been developed by a panel of German expert pulmonologists [22]. For each device, they included three major steps of inhalation: 1) inhalation preparation, 2) inhalation routine, and 3) closure of inhalation. After each demonstration, patients were asked to repeat the procedure shown for the device. If the inhaler technique was not yet correct, the investigator repeated the instructions for the respective device until the patient demonstrated error-free handling of the placebo inhaler. The number of attempts the patient needed to use the inhaler correctly was noted. To assess the correct inhaler technique, standardized checklists were used again [22].

After completion of the device training, all patients were asked for each of the 10 inhalers to assess device-related handling features using a 10-domain questionnaire. For this purpose, the following attributes were to be evaluated on a Likert scale, with response options from 5 (“applies perfectly”) to 1 (“not correct at all”): “I like the design of the device”, “It was easy to learn how to use the device”, “It was easy to prepare the device”, “The device is comfortable when held in my hand”, “The mouthpiece was comfortable when using the device”, “The device was easy to use”, “I felt that I had used the device correctly”, “I think it is possible to use the device easily and correctly in emergency situations”, “I am satisfied with the device”, and “Overall rating”. Finally, the participants were asked to name the device they would most and least prefer for everyday use, given comparable efficacy.

All interviews, device demonstrations and assessments were conducted by the same person to avoid misjudgement by different investigators. This whole assessment of 10 devices took between 90 and 120 min per patient.

### Statistical analysis

The program Microsoft Excel 2016 MSO (version 16.0.7329.1017), as well as WinSTAT for Microsoft Excel (version 2012.1.0.94) served as a basis for the statistical evaluation. Frequencies, mean values and standard deviations were calculated with WinSTAT.

The *p*-values for the number of attempts required until a device was used correctly were assessed using the Friedman test, followed by pairwise comparison using the Dunn test and a post-hoc correction according to Bonferroni. The same procedure was applied to evaluate device properties and handling features. Statistical significance was set at *p* < 0.05, whilst statistical trend was set at *p* < 0.10.

The significance of all other values was calculated using the Chi-square test and subsequent post-hoc Bonferroni correction. If the requirements for this test were not met, an exact Fisher’s test was performed.

## Results

### Patient characteristics and prescribed inhalers

One hundred-five patients participated in the study, including 61 patients with asthma and 44 with COPD. The average age of the test subjects was 56 years (SD = 17.4) and the mean disease duration was 12.7 years (SD = 10.9). Table 1 shows the characteristics of the patient population at the time of the survey.

**Table 1:** Characteristics of the patient population

N	105
Male, *n* (%)	45 (42.9)
Female, *n* (%)	60 (57.1)
Diagnosis, *n* (%)	
● Asthma -Males -Females ●COPD -Males -Females	●61 (58.1) -21 (34.4) -40 (65.6) ● 44 (41.9) -24 (54.6) -20 (45.4)
Patient age, mean (SD), years ●Asthma patients ●COPD patients	56.0 (17.4) ●47.4 (17.1) ●68.0 (8.53)
Age categories, *n* (%)	
●18–40 years of age ●41–60 years of age ●> 60 years of age	●24 (22.9) ●28 (26.7) ●53 (50.5)
Disease duration, mean (SD), years ●Asthma patients ●COPD patients	12.7(10.9) ●15.4(15.4) ●8.9 (8.0)

The 105 respondents used a total of 183 prescribed inhalers for their asthma or COPD therapy at the time of the survey (Table 2). The greater number of inhalers was due to the fact that some patients had more than one inhaler device available. Overall, pMDIs (39.34%) and the Turbohaler® (16.94%) were used most frequently and the Elpenhaler® (0.55%) and the Forspiro® (0.55%) the least. Among the group of asthma patients, pMDIs (46.53%) and the Turbohaler® (21.78%) were the most commonly used inhaler devices. COPD patients most often used a pMDI (30.49%) and the Breezhaler® (20.73%). The high percentage of pMDI users is partly explained by the fact, that pMDIs are frequently used as rescue medication. Furthermore, the distribution of devices used by the patients prior to the study also reflects prescription routines.

**Table 2:** Overview of prescribed inhaler devices used by 105 patients at the time of the survey

Inhaler device	Total number of patients, ***n*** (%)	Proportion within asthma patients, %	Proportion within COPD patients, %
Breezhaler®	18 (9.84)	0.99	20.73
Diskus®	9 (4.92)	7.92	1.22
Pressurized metered-dose inhaler (pMDI)	72 (39.34)	46.53	30.49
Elpenhaler®	1 (0.55)	0	1.22
Forspiro®	1 (0.55)	0	1.22
Genuair®	9 (4.92)	3.96	6.10
Nexthaler®	7 (3.83)	4.95	2.44
Respimat®	19 (10.38)	10.89	9.76
Spiromax®	3 (1.64)	0.99	2.44
Turbohaler®	31 (16.94)	21.78	10.98
None of the above	12 (6.56)	1.98	12.20
No device was currently used	1 (0.55)	0	1.22

**Fig. 1:**
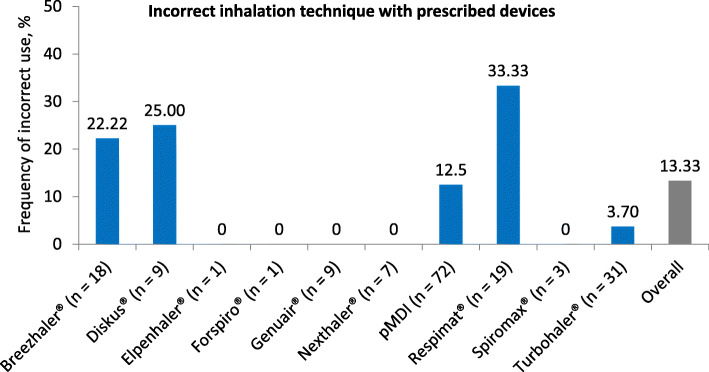
Incorrect inhalation technique with prescribed devices | Some patients made at least one critical error when demonstrating their inhalation technique with the device they were currently using. The figure shows what percentage of patients within the entire user group of the respective inhaler used their device incorrectly. The differences in the frequency of incorrect inhaler use did not reach statistical significance. n, patient number

86.67% of all subjects used their prescribed device correctly. However, 13.33% of the patients made at least one error while demonstrating their inhaler technique. Incorrect use was most frequently observed with the Respimat® and the Diskus® (p = n.s.; Fig. 1). When categorizing according to age groups, the highest error rate (20.37%) was found in patients over 60 years (*p* = 0.029 vs. younger age), compared with 0% in the 18–40 years of age group (*p* = 0.02 vs. other age groups).

77% of all patients stated that they had previously received an introduction to their current device. In 58, 15 and 4% of the cases, the instruction had been given by a doctor, a medical assistant / nursing staff or a pharmacist. 6% of the patients were unable to provide any information on this point and 17% of the patients stated that they had not received any instructions. The proportion of improperly used inhalers in the untrained group was 27.78%. Of the patients who received their instructions from the doctor, 13.33% made at least one error when using their prescribed inhaler. If the instruction had been given by a pharmacist or by medical assistants / nursing staff, the error rate was 0% (*p* = 0.059 vs. other goups).

**Fig. 2:**
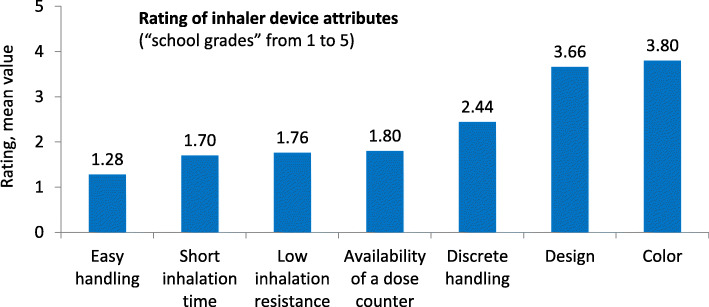
Rating of inhaler device attributes | Assessment of relevance of selected inhaler device properties from the perspective of asthma and COPD patients (*N* = 105), according to “school grades” from 1 to 5. The evaluation mean values for predefined inhalation properties are shown. The best possible rating was 1, the worst 5. N, patient number

When asked about the relevance of predefined inhaler attributes (“Which properties of an inhaler are important to you?”; best possible rating = 1), the patients stated easy handling as the most important characteristic (mean rating value: 1.28). This was followed by short inhalation time (1.70), low inhalation resistance (1.78) and availability of a dose counter (1.80). Least importance was attached to the design (3.66) and the color (3.80) of the device (Fig. 2).

### Error rates and patient ratings for 10 placebo devices

**Fig. 3:**
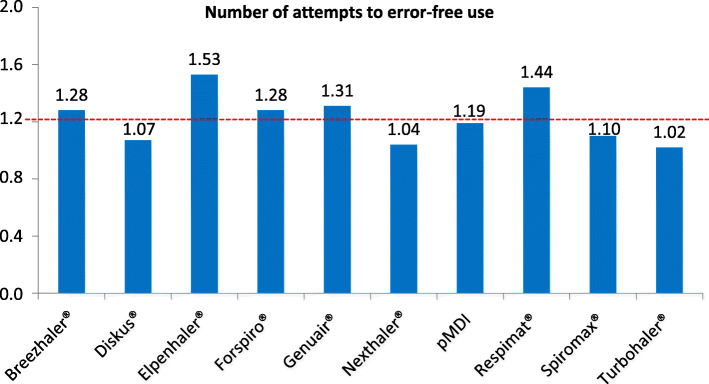
Number of attempts to error-free use | Number of attempts (mean) that patients needed until the inhaler device was used correctly. The dotted line shows the mean of attempts to error-free use across all 10 devices (1.22). None of the devices achieved a significantly lower error rate than others

Across all the presented and self-tested 10 placebo devices, patients needed an average of 1.22 attempts to use the inhaler correctly. The device with the lowest error rate was the Turbohaler® (1.02; p = n.s. vs. other devices). The Nexthaler® (1.04) and the Diskus® (1.07) followed in second and third place. The mean number of attempts until error-free use was highest with the Elpenhaler® (1.53; p = n.s. vs. other devices) and the Respimat® (1.44) (Fig. 3). 22.73% of COPD patients made at least one error in the handling of the placebo devices, compared to 6.56% in the group of asthma patients (*p* = 0.016). The group of 18- to 40-year-olds required the lowest number of attempts until the 10 placebo devices were used correctly (1.05; *p* = 0.005 vs. other age groups). The highest number of attempts was documented in patients over 60 years of age (1.33; *p* = 0.002 vs. other age groups).

**Fig. 4:**
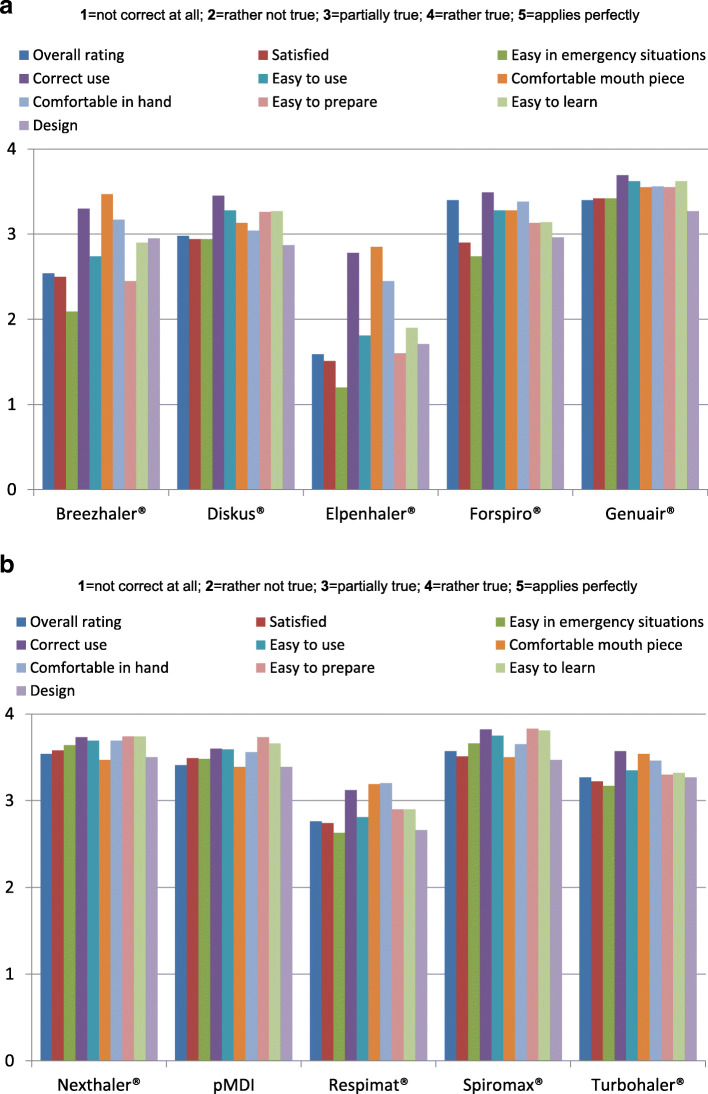
**a** Device-related handling characteristics, assessed by asthma and COPD patients. The best possible rating was 5, the worst 1. Design = “I like the design of the device“. Easy to learn = “It was easy to learn how to use the device“. Easy to prepare = “It was easy to prepare the device“. Comfortable in hand = “The device is comfortable when held in my hand“. Comfortable mouth piece = “The mouthpiece was comfortable when using the device“. **b** Device-related handling characteristics, assessed by asthma and COPD patients. The best possible rating was 5, the worst 1. Easy to use = “The device was easy to use“. Correct use = “I felt that I had used the device correctly“. Easy in emergency situations = “I think it is possible to use the device easily and correctly in emergency situations“. Satisfied = “I am satisfied with the device“

**Fig. 5:**
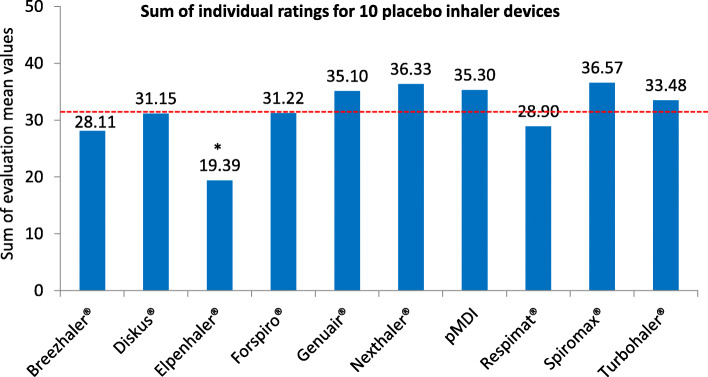
Sum of individual ratings for 10 placebo inhaler devices | Sum of the inhaler device ratings from Fig. 4**a** and **b**. The best possible rating was 50 points, the worst 10 points. The dotted line shows the evaluation mean sum across all 10 devices (31.56). *Elpenhaler vs. other devices *p* < 0.05

Figures 4a+b show for each of the 10 placebo devices how asthma and COPD patients assessed different device-related handling features. Response options ranged from 5 (“applies perfectly”) to 1 (“not correct at all”). When evaluating the device design, the test subjects rated the Elpenhaler® significantly worse than other devices (1.71; *p* < 0.05). The Nexthaler® achieved the best rating (3.50; p = n.s.). The Elpenhaler® also performed the worst (1.90; p < 0.05) in terms of ease of learning how to use the device. The Spiromax® achieved the best rating (3.81; p = n.s). With regard to ease of preparation of the device, the Elpenhaler® was rated significantly worse than other devices (1.60; p < 0.05). The Spiromax® achieved the best rating (3.83; p = n.s.), followed by the Nexthaler® (3.74). The test subjects stated that they were least able to hold the Elpenhaler® in their hands (2.45; p = n.s.). The best result in this category was achieved by the Nexthaler® (3.69; p = n.s.). The mouthpiece of the Elpenhaler® was rated worse than that of other devices (2.85; p = n.s.), while the mouthpiece of the Genuair® was felt to be the most comfortable (3.55; p = n.s.). The usability of a device was rated as significantly worse with the Elpenhaler® than with other devices (1.81; *p* < 0.05). The Spiromax® received the best rating for this domain (3.75; p = n.s.). With the Elpenhaler®, patients felt the least that they had used the device correctly (2.78; p = n.s.). The Spiromax® was rated as best (3.82; p = n.s.). The usability in emergency situations was assessed significantly worse with the Elpenhaler® than with other inhalers (1.20; *p* < 0.05). The Spiromax® received the best rating (3.66; p = n.s.). Overall satisfaction with an inhaler was rated worse for the Elpenhaler® than for other devices (1.51; *p* < 0.05). The Nexthaler® (3.58) received the best rating (p = n.s.). The Spiromax® (3.57; p = n.s.) and the Nexthaler® (3.54) achieved the best results in the overall assessment of all queried device properties. The total sum of the ratings of all 10 device properties was also highest for the Spiromax® (p = n.s.) and the Nexthaler® (Fig. 5).

### Inhaler device preferences

After demonstration and self-testing of all 10 placebo inhaler devices, 41% of the study participants chose one of the devices they already used in everyday life as a preference. This might have an impact on the further results. 7.6% opted for an inhaler they had used in the past and another 50.4% preferred an inhaler they had never used. Multi-dose dry powder inhalers (Diskus®, Forspiro®, Genuair®, Nexthaler®, Spiromax®, Turbuhaler®) were favored significantly more often than other inhaler devices (*p* = 0.017). This was true for both asthma patients (*n* = 48 vs. *n* = 13) and COPD patients (*n* = 27 vs. *n* = 17). Few patients opted for single-dose dry powder inhalers (*p* = 0.002 vs. other devices).

**Fig. 6:**
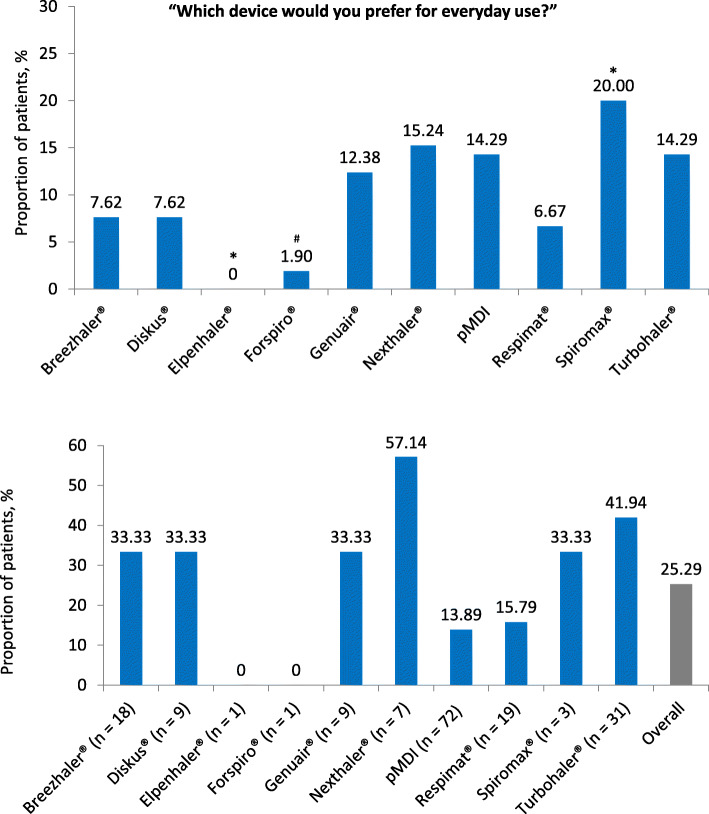
**a** Positive preference: device that patients (*N* = 105) would choose from amongst the 10 presented and self-tested placebo inhalers. Proportion of patients (%) who reported a positive preference for the respective device. Each patient was allowed to name only one device preference. N, patient number. *p = 0.006 vs. other devices. ^#^0.05 < *p* < 0.1 vs. other devices. **b** Percentage of patients who chose the device they were already using as the first preference, broken down by the 10 placebo inhalers tested

20% of the patients chose the Spiromax® as the inhalation device that they would most prefer (*p* = 0.006 vs. other devices; Fig. 6a). The Nexthaler® (15,24%) followed in second place and the Turbohaler® or a pMDI in third place (14,29% each). The least preferred device was the Elpenhaler® (0%; p = 0.006 vs. other devices). 5, 25, 66.67 and 86.67% of the patients who opted for the Spiromax®, the Nexthaler®, a pMDI or the Turbohaler®, respectively, were already using the device in their everyday life. Figure 6b shows, for each of the 10 test devices, the proportion of patients who, when asked about the preference for an inhaler, chose the device already known and prescribed for them.

Patients with asthma named the Spiromax® (19.67%), the Turbohaler® (19.67%; *p* = 0.046 vs. COPD patients) and the Nexthaler® (18.03%) as the most preferred devices. In the group of COPD patients, these were the Spiromax® (20.45%), the Breezhaler® (18.18%; *p* < 0.001 vs. asthma patients) or a pMDI or the Genuair® (13.64% each). Women preferred the Spiromax® more often than men (*p* = 0.006), while more men than women chose the Nexthaler® as their first preference (p = n.s.). Subjects between the ages of 18 and 40 most often preferred a pMDI or the Nexthaler® (25.00% each; p = n.s. vs. other devices). In the age group of 41 to 60-year-olds, most patients chose Spiromax® (29.63%; p = n.s.). Patients over the age of 60 most frequently named the Turbohaler® as their preferred device (19.00%; p = n.s.).

## Discussion

Each type of inhaler device is associated with advantages and limitations that determine their suitability for any given patient with asthma or COPD. Understanding those advantages and limitations may help clinicians in choosing the proper device for the individual patient. A general goal is to improve patient compliance and achieve the best possible treatment results. It is therefore important to tailor the selection of the inhaler device to the individual patient, taking into account their needs and preferences [15, 17, 23–25].

The aim of this industry-independent cross-sectional study was to examine the error rates in inhaler technique with a selection of commonly prescribed inhalers and to find out which devices are preferred by patients with asthma or COPD, taking into account age and gender.

### Error rates with prescribed inhalers

In our study, patients using their own inhaler showed only a moderate error rate of 13.33%. Other working groups, however, reported higher error rates of up to 80% [26]. This divergence could be due to different assessment methods, evaluation periods and inhalers used, which makes it difficult to compare results. The patients we examined were most often prescribed a pMDI or the Turbohaler®. They rated the use of these devices as relatively simple. On the other hand, devices whose handling is classified as more difficult (e.g. Elpenhaler®) were used by only very few subjects. Some earlier studies reported a higher device handling error rate in female patients [14], others, however, could not [2]. Our study found no significant gender differences in error rates. However, the error rate was higher in patients with COPD and in the elderly over 60 years. It must be taken into account that the average age in COPD patients is generally higher than in asthma patients.

Especially patients without device training had higher error rates with their own inhaler. Special attention should be therefore paid to instructing patients on their device [27]. Studies have shown that repeated, continuous and interactive training is particularly promising [28]. In our study, patients made fewer errors if the training was given by a pharmacist or by medical assistant / nursing staff than when the training was done by a doctor. This result showed a trend towards statistical significance. Nevertheless, the number of patients, which were trained by a medical assistant or parmacist was to small to draw valid conclusions, but this observations might indicate, that these professions may play a relevant role in structured patient care.

### Patient assessment of 10 placebo devices

The evaluation of the error rate with the placebo devices was given in our study as “number of attempts until error-free use”. Other studies apply a variety of different methods, such as: rate of steps performed wrong [14], number of critical errors [2], number of errors in key actions essential to the delivery of active drug [29], or awarding a “school grade” for inhaler technique [30]. These differences in assessment methods make it difficult to compare error rates in inhaler technique between studies. In our study, the test subjects needed an average of 1.22 attempts before the demonstrated placebo devices could be used without errors. No significant gender difference was found. COPD patients and the elderly had more attempts to use the inhaler devices correctly. Patients needed the least number of attempts when inhaling with the Turbohaler® or the Nexthaler®, but these results did not achieve statistical significance. Most attempts were documented with the Elpenhaler® and the Respimat®.

Research confirms that there are substantial differences in patient’s preference and acceptability for inhalers, mainly related to the handling of the different devices [31]. All 105 patients were asked to rate the handling of the 10 placebo inhalers based on predefined questions. The Spiromax®, the Nexthaler® and a pMDI received the highest ratings across all domains. The Elpenhaler®, the Breezhaler® and the Respimat® had the lowest rating. None of the devices achieved a significantly higher total sum score than others.

For daily use, 20% of the patients preferred the Spiromax® and more than 15% the Nexthaler®. While asthma patients preferred the Spiromax®, the Turbohaler® and the Nexthaler® more often, in COPD patients these were the Spiromax®, the Breezhaler® and a pMDI or the Genuair®. The majority of patients named the Elpenhaler® as the least favored device for everyday use. This agrees with the overall poor ratings of the individual inhaler properties and the higher number of attempts required until correct use of this device. Other study groups also found a rather low patient preference for the Elpenhaler [32]. Overall, multi-dose dry powder inhalers have been mentioned more often as a first preference than single-dose powder inhalers. This observation corresponds to the results of other study groups [13]. It is also known from the literature that preferences seem to be greatly influenced by the prescription experience of the patients [14]. Chorão et al. found that 66% of asthma or COPD patients chose the easiest device and 49% the preferred for routine use among those currently or formerly used [14]. Our study confirms that about four out of 10 patients opted for a device that they already used in everyday life.

Device handling, correct inhaler technique, patient preference, and adherence are intertwined factors that may all contribute to good symptom control [24]. Other studies witness that devices with the lowest number of handling errors had the highest ratings in patient preferences [33], suggesting that a patient’s acceptance of a device may be correlated with ease of handling [34]. Accordingly, study groups identified the ease of use of a device as one of the most important features for an ideal inhaler [35]. It is important for patients that the instructions for operating the inhaler are easy and simple to follow [15]. A study conducted in France assessed asthma and COPD patients’ preferences for different attributes of DPIs. Here, as well, patients placed highest values on attributes related to ease of use [8]. Ding et al. examined inhaler preferences in asthma and COPD from the patient’s perspective, particularly focusing on the relative importance of individual device attributes and patient characteristics guiding inhaler choice [15]. Instructions being simple and easy to follow was the inhaler attribute most commonly selected as important [15]. This can be reproduced in our study. The asthma and COPD patients confirmed an easy handling as the most important feature for them. This was followed by a short inhalation time and a low inhalation resistance.

### Limitations

We prospectively collected data from a sample of hospitalized and ambulant asthma and COPD patients. Our study offers insight into eliciting possible patient preferences for inhaler devices. Nevertheless, the results should be interpreted within the context of study limitations.

This is a cross-sectional clinical study. This means that the data were collected at a single point in time. As a result, the study gives a less comprehensive impression than a longer-term observation. Based on our results, no statement can be made as to whether the error rates in device use or inhaler preferences change over time. Because correct handling of an inhaler prescription is usually only checked once, but not repeatedly, by a healthcare professional, the design we chose may most closely reflect real life.

Both inpatient and outpatient treatments were included. Since this is a monocentric study, a bias in patient recruitment cannot be ruled out, and results may not be extrapolated to other regions and populations.

Our study examined widely used, but not all, inhalers available on the German market. With a total of 10 devices, a large number of different inhalers was tested in order to get the most comprehensive picture possible. However, this also meant that patients had to answer a variety of questions. Though randomizing the demonstration and testing order of the devices, signs of fatigue in the test subjects with the devices queried later cannot be ruled out and may have resulted in less variance in results between devices.

Asthma and COPD patients were not naive to inhalation therapy. It cannot be excluded that previous experience with the inhalers tested here or with other inhalers has influenced and biased the assessment and preference for certain devices.

The primary benefit of DPIs, when compared with pMDIs and soft mist inhalers, is medication delivery through breath actuation, therefore decreasing issues related to patient synchrony. However, optimal peak inspiratory conditions are required to actuate the device as well as properly deliver the medication [36]. Therefore, it would be beneficial to know how dry-powder inhaler design interacts with the airway dynamics of patients [37, 38]. This parameter was not considered in our study.

## Conclusion

Particular attention should be paid to choosing the appropriate inhaler device for asthma and COPD patients, respectively. Our industry-independent study included inhalers that are widely used in Germany (Breezhaler®, Diskus®, Elpenhaler®, Forspiro®, Genuair®, Nexthaler®, a customary pMDI, Respimat®, Spiromax® and Turbohaler®). The Spiromax® and the Nexthaler® turned out to be the most popular devices in the total patient group. As a first preference, around four out of 10 patients chose a device that they already used in daily routine. Multi-dose dry powder inhalers were generally more preferred than single-dose DPI, a pMDI or the soft mist inhaler.

The choice of the most suitable inhaler is a complex decision taken between doctor and patient. Important inhaler attributes from the patient’s point of view are, above all, ease of use, a short inhalation time and a low inhalation resistance. Identifying patient preferences for an inhaler device may further increase patient adherence to therapy and thus improve disease outcome. Prescription of an inhaler device should therefore not be standardized and each patient must be considered individually.

## Data Availability

The datasets used and/or analysed during the study are available from the corresponding author on reasonable request.

## Abbreviations

COPD: Chronic obstructive pulmonary disease
DPI: Dry-powder inhaler
FSI-10: Feeling of Satisfaction with Inhaler questionnaire
N/n: Number
n.s.: Not significant
PASAPQ: Patient Satisfaction and Preference Questionnaire
pMDI: Pressurized metered-dose inhaler
SD: Standard deviation
vs.: Versus
